# High Glucose-Induced Kidney Injury via Activation of Necroptosis in Diabetic Kidney Disease

**DOI:** 10.1155/2023/2713864

**Published:** 2023-01-30

**Authors:** Man Guo, Qing Chen, Yongli Huang, Qi Wu, Yan Zeng, Xiaozhen Tan, Fangyuan Teng, Xiumei Ma, Yueli Pu, Wei Huang, Junling Gu, Chunxiang Zhang, Yang Long, Yong Xu

**Affiliations:** ^1^Department of Endocrinology and Metabolism, The Affiliated Hospital of Southwest Medical University, Luzhou 646000, China; ^2^Metabolic Vascular Disease Key Laboratory of Sichuan Province, The Affiliated Hospital of Southwest Medical University, Luzhou 646000, China; ^3^Department of Outpatient, The Affiliated Hospital of Southwest Medical University, Luzhou 646000, China; ^4^Department of Pathology, and Academician Workstation of Sichuan Province, The Affiliated Hospital of Southwest Medical University, Luzhou 646000, China; ^5^Faculty of Chinese Medicine, Macau University of Science and Technology, Avenida Wai Long, Macau, China; ^6^Department of Endocrinology, Yibin Second People's Hospital, Yibin 644000, China; ^7^Institute of Cardiovascular Research, Southwest Medical University, Luzhou 646000, China; ^8^Experimental Medicine Center, The Affiliated Hospital of Southwest Medical University, Luzhou 646000, China

## Abstract

Diabetic kidney disease (DKD) is a major microvascular complication of diabetes mellitus (DM) and is closely associated to programmed cell death. However, the complex mechanisms of necroptosis, an alternative cell death pathway, in DKD pathogenesis are yet to be elucidated. This study indicates that necroptosis is involved in DKD induced by high glucose (HG) both in vivo and in vitro. HG intervention led to the activation of RIPK1/RIPK3/MLKL signaling, resulting in renal tissue necroptosis and proinflammatory activation in streptozotocin/high-fat diet- (STZ/HFD-) induced diabetic mice and HG-induced normal rat kidney tubular cells (NRK-52E). We further found that in HG-induced NRK-52E cell, necroptosis might, at least partly, depend on the levels of reactive oxygen species (ROS). Meanwhile, ROS participated in necroptosis via a positive feedback loop involving the RIPK1/RIPK3 pathway. In addition, blocking RIPK1/RIPK3/MLKL signaling by necrostatin-1 (Nec-1), a key inhibitor of RIPK1 in the necroptosis pathway, or antioxidant N-acetylcysteine (NAC), an inhibitor of ROS generation, could effectively protect the kidney against HG-induced damage, decrease the release of proinflammatory cytokines, and rescue renal function in STZ/HFD-induced diabetic mice. Inhibition of RIPK1 effectively decreased the activation of RIPK1-kinase-/NF-*κ*B-dependent inflammation. Collectively, we demonstrated that high glucose induced DKD via renal tubular epithelium necroptosis, and Nec-1 or NAC treatment downregulated the RIPK1/RIPK3/MLKL pathway and finally reduced necroptosis, oxidative stress, and inflammation. Thus, RIPK1 may be a therapeutic target for DKD.

## 1. Introduction

Type 2 diabetic kidney disease (DKD) is a major microvascular complication of diabetes mellitus (DM), which is the leading cause of chronic renal disease (CKD) and end-stage renal disease (ESRD) worldwide [1, 2]. The mechanisms leading to the initiation and progression of renal dysfunction in DKD are yet to be elucidated. Confounding factors have been reported that mainly relate to metabolic disorders, inflammatory responses, oxidative stress [3], and DNA methylation profiles [4]. Cell depletion, loss or necrosis in the form of apoptosis, and other programmed cell death pathways may also play important roles and are recognized as considerable drivers of progressive decline in renal function [5, 6]. Both apoptosis and necrosis can cause tubular injury. Renal cell loss which is partly a consequence of apoptosis is the predominant mechanism mediating renal tubular epithelial cell loss and is central to the pathophysiology of renal damage [7–9].

Necroptosis shares several upstream signaling pathways with apoptosis; cell death can switch into a specific form of necrosis when aspartate-specific cysteine protease-8 (caspase-8) is defective, resulting in necroptosis [10, 11]. Necroptosis, a recently recognized form of nonapoptotic, regulated necrotic cell death, has a typical necrotic morphology. It can be activated by death receptors such as tumor necrosis factor receptor 1 (TNFR1) and a factor associated with suicide (FASL) [12, 13]. Among the various triggers, TNF-*α*/TNFR signaling has been the most typical and intensively investigated [14]. The binding of TNF to TNFR1 leads to the activation of receptor-interacting protein kinase 1 (RIPK1) and receptor-interacting protein kinase 3 (RIPK3), resulting in the recruitment a RIPK1-RIPK3-mixed lineage kinase domain-like protein (MLKL) complex that is localized on the cell membrane via phosphorylation of MLKL by RIPK3. This leads ultimately to the disruption of the plasma membrane and causes cell lysis [15, 16]. Reactive oxygen species (ROS), DNA methylation profiles, mitochondrial bioenergetic disorders, and advanced glycation end products (AGEs) have been implicated as necrotic effectors [17]. RIPK1, a crucial regulator of cell fate [18], functions as a start switch for caspase-8-dependent apoptotic or RIPK3/MLKL-mediated necroptosis, as well as for the activation of the nuclear factor-*κ*B (NF-*κ*B) pathway that is involved in promoting cell survival and inflammation [19]. RIPK1 can be regulated and pharmacologically inhibited by necrostatin-1 (Nec-1). Nec-1 is a cellular protector that has been widely used in various cellular and animal models of human diseases and is especially sensitive to TNF-induced necroptosis [20–22].

Accumulating evidence has shown that necroptosis plays an important role in various pathological conditions in humans, including neurodegenerative and cerebrovascular diseases [23–25], liver and retinal injuries [21, 26], and osteoporosis [27, 28]. Few studies have reported the role of necroptosis in the pathogenesis of renal diseases. Although necrosis nephron segment injury is the main mediator of acute kidney injury (AKI), necroptosis is another alternative cell death pathway that operates alongside [29]. Necroptosis is the primary mechanism mediating renal tubular epithelial cell loss in early and intermediate chronic renal disorders that result from necrosis preceding regeneration and/or fibrotic tissue remodeling [30]. Diabetes-related risk factors such as high glucose, AGEs, and lipopolysaccharide (LPS) can induce necroptosis in cardiomyocytes [31, 32], podocytes [33], glomerular endothelial cells, and umbilical vein endothelial cells [34]. It was speculated that high glucose-induced necroptosis mediated by TNF might be further regulated by ubiquitin carboxy-terminal hydrolase L1 (UCHL1) via the RIPK1/RIPK3 pathway, leading to enhanced progression of diabetic nephropathy and increased podocyte injury and loss [33]. Nec-1 effectively protects against renal ischemia and reperfusion (I/R) injury by inhibiting necroptosis and oxidative stress [35]. However, whether RIPK1 mediates damage by high glucose stimulation via activation of necroptosis in DKD patients and animal models remains unknown. Moreover, it has not yet been investigated whether necrostatin-1 effetely repairs the injury and ameliorates loss of renal tubular epithelial cells and if the antioxidants of N-acetylcysteine (NAC) decrease the release of inflammatory cytokines by inhibiting the RIPK1/RIPK3 pathway.

To validate this hypothesis, we created an in vitro cell model of HG intervention using normal rat kidney tubular cells (NRK-52E) and an in vivo mouse model of DKD along with an investigation of human kidney tissue samples collected postsurgery. Our research revealed that HG treatment increased renal tubular epithelial cell necroptosis and accelerated renal injury and fibrosis and that ROS are a driving force for necroptosis to an extent. Furthermore, Nec-1 and NAC were found to ameliorate renal function via inhibition of the RIPK1/RIPK3/MLKL signaling pathway. Our findings indicate that RIPK1 and targeted antioxidants may be potential therapeutic targets for DKD.

## 2. Materials and Methods

### 2.1. Kidney Sample Collection from Patients

Three patients with DKD (aged 56–65 years, two males and one female) in the Department of Pathology and three nondiabetic patients in the Department of Urology, Affiliated Hospital of Southwest Medical University, China, were recruited for histological and immunohistochemical studies. DKD was diagnosed by the urinary microalbumin/creatinine ratio (UACR, >30 mg/g) and typical pathological manifestations of the kidney, in accordance with the policies of the Clinical Trial Ethics Committee of the Affiliated Hospital of Southwest Medical University (KY2021086).

### 2.2. Animal Experiments

The animal experimental protocols were approved by the Animal Research Center of Southwest Medical University (20210928-007) and were performed in compliance with the policies of the Chinese Animal Research Committees. Six-week-old male C57BL/6J mice were purchased from Chengdu Dossy Experimental Animal Co., Ltd. (China) and randomly assigned to four groups: control vehicle-treated (NC), necrostatin-1 drug control (Nec-1), diabetic kidney disease (DKD), and type 2 diabetes mellitus (T2DM) injected with necrostatin-1 (DKD+Nec-1) (*n* = 15/group). After feeding the mice a high-fat diet for 8 weeks and followed by the occurrence of insulin resistance, 50 mg/kg streptozotocin (STZ, Sigma-Aldrich, Louis, USA) was continuously injected intraperitoneally (ip) for 4 days. When the fasting blood glucose level was higher than 16.7 mM, 1.65 mg/kg/d of necrostatin-1 (Sigma-Aldrich, Louis, USA) was injected intraperitoneally, and the control received 1000 *μ*L/kg/d of 1‰ DMSO solution (Sigma-Aldrich, Louis, USA) in the same volume for 16 weeks.

Mice were anesthetized with 1% pentobarbital sodium (50 mg/kg body weight, ip, Sigma-Aldrich, Louis, USA), and blood was collected for the detection of the blood urea nitrogen (BUN) levels and urine albumin-to-creatinine ratio (ACR) and urine for microalbuminuria. Microalbuminuria was measured by ELISA, ACR by picric acid colorimetry, and BUN by urease. The kidneys were cut along the coronal plane, and the right was used for pathological assessments and the left for western blotting and real-time PCR assays.

### 2.3. Cell Culture

The NRK-52E cells were purchased from American Type Culture Collection (ATCC, USA) and cultured in Dulbecco's modified Eagle's medium (DMEM; Gibco, Grand Island, NY, USA) containing 5.6 mM glucose and supplemented with 10% fetal bovine serum (FBS; Gibco, Grand Island, NY, USA), 100 U/mL penicillin, and 100 *μ*g/mL streptomycin (Invitrogen, Grand Island, NY, USA). The incubated cells were grown at 37°C with 5% CO_2_ and were starved for 6 h and treated with different concentrations of glucose (5.6 mM for control group and 30 mM for high glucose intervention) and then pretreated with 50 *μ*M Nec-1 and 2 mM NAC for 24, 48, and 72 h. Following induction for 48 h, the total protein and mRNA were extracted from the cells for further study.

### 2.4. Cell Viability Assay

Cell growth and viability were measured using 3-(4,5-dimethylthiazol-2-yl)-2,5-diphenyltetrazolium bromide (MTT; Beyotime Institute of Biotechnology, Shanghai, China) assay. NRK-52E cells (1 × 10^5^ cells/well) were seeded in 96-well plates in growth medium and pretreated with different concentrations of glucose; 10 *μ*L of MTT (1 mg/mL) was added to each well, with 5 replicate wells, to allow the formation of MTT formazan crystals at 37°C for 4 h, which were solubilized in 100 *μ*L of DMSO. Cell proliferation was recorded at 570 nm (Thermo Fisher Scientific, MA, USA) according to the manufacturer's instructions, and the appropriate time and concentration of glucose intervention were determined. The same method was used for Nec-1 and NAC.

### 2.5. Histopathological Analysis

Human and mouse kidneys were fixed in 4% PFA for 24 h and embedded in paraffin. The sections were deparaffinized, dehydrated, and stained with hematoxylin and eosin (H&E) and subjected to Masson's staining for histomorphometric analysis (Leica, Germany). Other sections subjected to immunofluorescence and immunohistochemical (IHC) analysis were incubated with rabbit phosphor-RIPK1 (p-RIPK1; 1 : 100; Cell Signaling Technology, USA), mouse phosphor-RIPK3 (p-RIPK3; 1 : 100; Abcam, Cambridge, UK), phospho-MLKL (p-MLKL; 1 : 100; Abcam, Cambridge, UK) antibodies, and anti-active caspase-3 (1 : 100; Abcam, Cambridge, UK), treated with goat anti-rabbit IgG (1 : 100), and incubated with streptavidin-horseradish peroxidase complex (HRP, 1 : 100, Biosynthesis Biotech, China). To visualize the signals, the sections were treated with the peroxidase substrate DAB (3,3-diaminobenzidine) and counterstained with hematoxylin. The renal tissue structure was observed, and the percentage of p-RIPK1-, p-RIPK3-, and p-MLKL-positive cells was calculated using Image-Pro Plus software.

### 2.6. Western Blotting Analysis

Total protein was extracted from kidney and mouse NRK-52E cells using the RIPA cell lysis buffer system (Cell Signaling Technology, USA), supplemented with phosphatase inhibitors, and was quantified by BCA-protein assay kit (Beyotime Institute of Biotechnology, Shanghai, China). An aliquot (20 *μ*g) of the proteins was separated by 10% SDS-PAGE for protein electrophoresis and transferred to a polyvinylidene fluoride (PVDF) membrane (Millipore). Following blocking in 5% nonfat dry milk for 1 h at room temperature, the membrane was incubated at 4°C overnight with primary antibodies. The following antibodies were used: mouse RIPK1, p-RIPK3/RIPK3, p-MLKL/MLKL, IL-1*β*, anti-active caspase-3, phosphor-inhibitor *κ*B *α* (p-I*κ*B*α*), phosphor-inhibitor of kappa B kinase *α*/*β* (p-IKK*α*/*β*), GAPDH (all antibodies dilution ratios are 1 : 1000; Abcam, Cambridge, UK), and rabbit p-RIPK1. After washing three times with phosphate-buffered saline with Tween-20 (PBST), the membrane was incubated with HRP-conjugated secondary antibody (1 : 2000, Abcam, Cambridge, UK) at room temperature for 1 h. Bands were quantified using ImageJ software and normalized to GAPDH.

### 2.7. Quantitative Real-Time PCR (qRT-PCR) Analysis

Total RNA was isolated from mouse kidney tissue and NRK-52E cells using TRIzol reagent (Qiagen, Valencia, CA, USA), and the concentration and purity were assessed using a spectrophotometer. The isolated RNA was subjected to reverse transcription using a ReverTra Ace® qPCR RT Kit (Toyobo, Japan), and the synthesized cDNA was used as a template for quantitative PCR analysis. The primer sequence of monocyte chemoattractant protein- (MCP-) 1 was determined using Primer Premier 5.0 software, which confirmed the definition of primers in the NCBI website via Primer-BLAST, and synthesized by Shanghai Biotechnology Co., Ltd. Quantitative PCR reactions were performed in triplicate to remove any outliers. Finally, the CT values were analyzed in relation to GAPDH CT values (RQ = −ΔΔCt).

### 2.8. Intracellular ROS Assay, ELISA Assay, and TUNEL Assay

The intracellular production of ROS was measured using a ROS detection kit (Beyotime Institute of Biotechnology, Shanghai, China). The fluorescence of 2′-7′-dihydrodichlorofluorescein diacetate (DCFH-DA) was determined using a spectrofluorophotometer (BMG LABTECH, Germany) and measured in a plate reader with excitation at 488 nm and emission at 525 nm according to the manufacturer's instructions. Urine microalbuminuria levels were measured using a mouse microalbuminuria ELISA kit (Beijing Cheng Lin Biological Technology Co. Ltd., China) according to the manufacturer's protocols in 96 wells. The same method was applied to examine urine creatinine, BUN, and ACR in mouse serum. Apoptosis was detected using a TUNEL assay. Sections were incubated with 50 *μ*L TUNEL reaction mixture in a wet box for 60 min at 37°C in the dark. Apoptotic cell death was quantified using a fluorescence microscope in the wavelength range of 570-620 nm (Roche).

### 2.9. Data Analysis

All data are expressed as mean ± standard deviation (SD) from at least three independent experiments, and comparisons between multiple groups were analyzed using one-way analysis of variance (ANOVA) with SPSS 22.0 software (SPNN Inc., Chicago, IL, USA). Statistical significance was set at *p* < 0.05.

## 3. Results

### 3.1. Protein Signaling Necroptosis Increased in Human DKD

**Figure 1:**
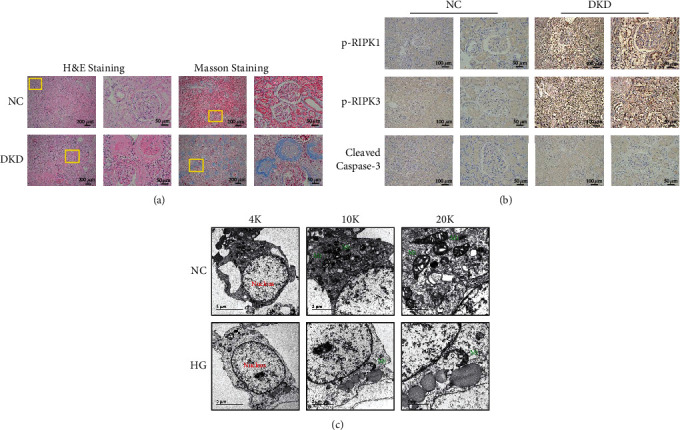
Necroptosis found in the kidney of DKD patients. (a) H&E and Masson's staining showing the pathological renal structure and deposited collagenous matrix in DKD patients. Scale bar: 200 *μ*m and 50 *μ*m, respectively. (b) IHC staining showing the difference in the expression of p-RIPK1 and p-RIPK3, and not of anti-active caspase-3, and the positive expression on renal tubules in DKD. Scale bar: 100 *μ*m. (c) TEM analysis reveals that NRK-52E cells exhibit normal nuclear and cytoplasmic morphology in the control group and display typical necrotic ultrastructural changes including a disrupted plasma membrane, fragmented and vacuolated mitochondrial membranes (green mark), and expanded endoplasmic reticulum after exposure to 30 mM HG for 48 h. Scale bar: 5 *μ*m and 1 *μ*m, respectively. p-RIPK1: phosphor receptor-interacting serine/threonine kinases 1; p-RIPK3: phosphor receptor-interacting serine/threonine kinases 3; TEM: transmission electron microscopy; Mt: mitochondrion.

To explore cell lysis by necroptosis in DKD, we performed IHC analysis for the necroptosis markers p-RIPK1 and p-RIPK3 in kidney biopsy specimens from three nondiabetic patients (control, NC) and three patients with DKD. Histomorphometric analysis performed on H&E-stained sections revealed that glomerular volume decreased and glomerular basement membrane (GBM) thickness increased in diabetic patients. It also indicated glomerulosclerosis and mesangial thickening in these patients. Masson's staining showed collagenous matrix deposition in glomeruli and tubules (indicated by more collagen deposition, blue staining in Figure 1(a)) and tubulointerstitial fibrosis. These are the typical renal pathological manifestations of DKD. Notably, IHC staining indicated an increase in the expression of p-RIPK1 and p-RIPK3 in glomeruli, especially in renal tubules, whereas the cleaved caspase-3 levels were not significantly different between the NC and DKD groups (Figure 1(b)). Therefore, we focused on the necroptosis of renal tubules for the remaining study. Next, NRK-52E cells were cultured and stimulated by high glucose (30 mM) for 48 h. Transmission electron microscopy (TEM) analysis revealed that the plasma membrane was disrupted, mitochondrial membranes fragmented and vacuolated, and the endoplasmic reticulum expanded (Figure 1(c)), which are the typical characteristics of necroptosis. We concluded that the renal tubules developed necroptosis in patients with DKD.

### 3.2. HG-Induced Necroptosis of Renal Tubular Epithelial Cells by the Activation of RIPK1/RIPK3/MLKL Signaling

**Figure 2:**
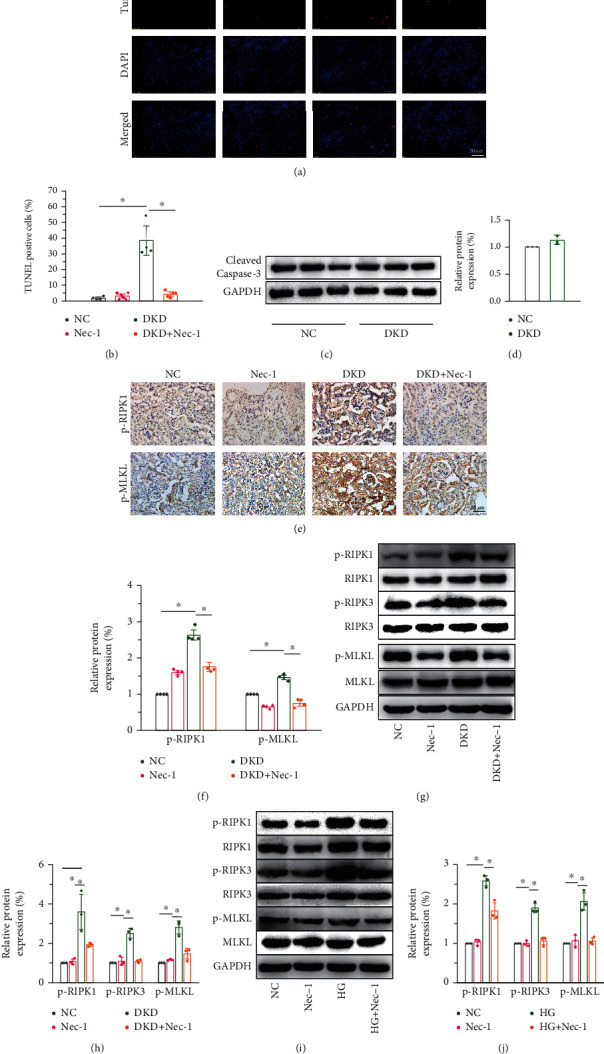
HG-induced necroptosis in kidney by activation of the RIPK1/RIPK3/MLKL signaling in diabetic mice. (a, b) TUNEL staining indicating the increase in necrotic cells in STZ/HFD-induced diabetic mice. Scale bar: 50 *μ*m. (c, d) No significant difference is seen in the protein levels of cleaved caspase-3 between the NC and DKD groups. (e, f) IHC analysis revealing that the key markers of necroptosis (p-RIPK1 and p-MLKL) are significantly elevated in diabetic mice. Scale bar: 50 *μ*m. Western blot confirming the upregulation of p-RIPK1 and p-RIPK3 and the downstream effector, p-MLKL, in vivo (g, h) and in vitro (i, j). Nec-1 treatment of 1.65 mg/kg/d for 16 weeks in mice and 50 *μ*M for 48 h in cells, respectively. All experimental data were verified in at least three independent experiments. Error bars represent the SD from the mean values. ^∗^*p* < 0.05. p-MLKL: phosphorylated mixed lineage kinase domain-like protein; Nec-1: necrostatin-1.

To investigate the effects of HG-induced necrocytosis in the mouse model of streptozotocin/high-fat diet- (STZ/HFD-) induced DKD, we detected necroptosis signaling. Interestingly, TUNEL staining indicated that the number of necrotic cells increased in the DKD kidneys (Figures 2(a) and 2(b)). The apoptosis marker, anti-active caspase-3, showed no significant change in the kidneys of mice with HG-induced DKD (Figures 2(c) and 2(d)). HG markedly elevated necroptosis in renal tissue, as indicated by the increased number of p-RIPK1- and p-MLKL-positive cells (Figures 2(e) and 2(f)) observed by IHC staining. Further, to investigate the molecular signaling pathways underlying cell death, we cultured NRK-52E cells, starved them for 6 h, and stimulated them with 30 mM glucose, followed by a 50 *μ*M Nec-1 treatment for 48 h each. The protein expression levels of p-RIPK1, p-RIPK3, and p-MLKL increased correspondingly, and Nec-1 treatment lowered these elevated levels both in vivo (Figures 2(g) and 2(h)) and in vitro (Figures 2(i) and 2(j)). Therefore, we concluded that high glucose-induced cell death could mainly be attributed to necroptosis rather than apoptosis of renal tissue and NRK-52E cells and depends on the activation of RIPK1/RIPK3/MLKL signaling.

### 3.3. Nec-1 Treatment Ameliorated Renal Dysfunction and Pathophysiology in DKD Mice

**Figure 3:**
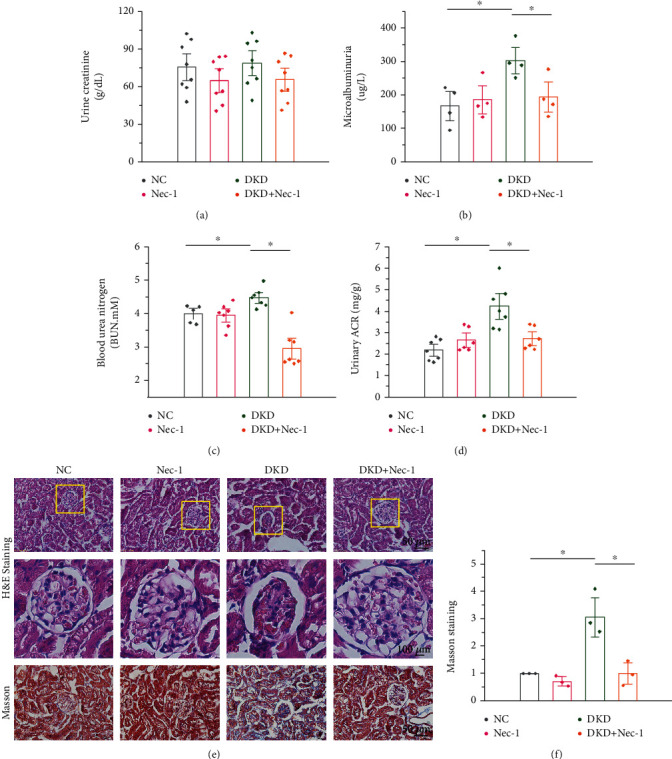
Nec-1 treatment ameliorates renal dysfunction in DKD mice. (a–d) Nec-1 (1.65 mg/kg/d for 16 weeks) treatment substantially reduces the increased urine microalbuminuria, blood urea nitrogen (BUN), and urine albumin-to-creatinine ratio (ACR) in mice serum as detected by ELISA. (e) H&E-stained sections revealing that Nec-1 treatment significantly improves the pathological changes in DKD, such as mesangial cell proliferation, matrix increase, basement membrane thickening, and increase in interstitial inflammatory cells. Scale bar: 50 *μ*m and 100 *μ*m. (f) Masson's staining showing the renal fibrosis in mice with DKD improved significantly after treatment with Nec-1. Scale bar: 50 *μ*m.

Necroptosis occurs in the renal tissues of STZ/HFD-induced diabetic mice, and RIPK1 plays an important role in the occurrence and development of DKD. To examine whether necrostatin-1 treatment ameliorated renal dysfunction, we treated mice with 1.65 mg/kg/d Nec-1 for 16 weeks and observed renal function, morphological changes, and fibrosis. We found that Nec-1 treatment substantially reduced the increased urine microalbuminuria, BUN, and ACR, as assayed by ELISA (Figures 3(a)–3(d)). Furthermore, H&E staining revealed that Nec-1 treatment significantly improved pathological changes in the kidney and inhibited mesangial cell proliferation and decreased matrix increase, basement membrane thickening, and interstitial inflammatory cell infiltration (Figure 3(e)). Masson's staining showed that the renal fibrosis in DKD mice was significantly ameliorated following Nec-1 treatment (Figures 3(e) and 3(f)). Collectively, these data suggest that Nec-1 significantly improves renal function, renal remodeling, and fibrosis in DKD mice.

### 3.4. Nec-1 Treatment Reduced Kidney Inflammation Induced by HG

**Figure 4:**
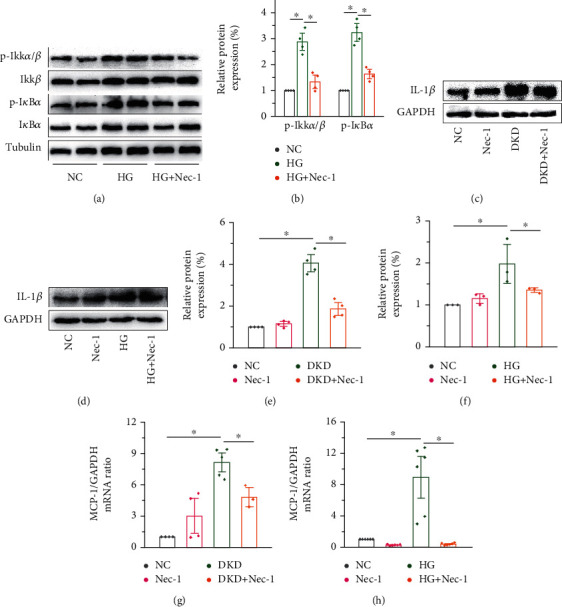
Nec-1 treatment reduces kidney inflammation induced by HG. (a, b) The protein expression levels of p-I*κ*B*α* and p-IKK*α*/*β* increased correspondingly, and Nec-1 effectively lowered the elevated levels. The expression levels of IL-1*β* were upregulated after HG intervention; however, Nec-1 treatment (1.65 mg/kg/d for 16 weeks in mice and 50 *μ*M for 48 h in cells, respectively) downregulated the expression levels both in vivo (c, e) and in vitro (d, f). qRT-PCR showing the significant increase in the expression of MCP-1 in DKD mice (g) and cells in the high glucose group (h), and a clear decrease following the use of RIPK1 inhibitor Nec-1. MCP-1: monocyte chemotactic protein-1.

Immune-mediated chronic low-grade inflammation is closely associated with the pathogenesis of diabetes mellitus and microvascular complications. Inflammatory cytokines are involved in the progression of diabetic nephropathy [36]. The protein expression levels of p-I*κ*B*α* and p-IKK*α*/*β* correspondingly increased, and Nec-1 effectively suppressed this increase (Figures 4(a) and 4(b)). IL-1*β* expression was upregulated after HG intervention, both in vivo (Figures 4(c) and 4(e)) and in vitro (Figures 4(d) and 4(f)). The expression of MCP-1 mRNA was significantly increased in DKD mice (Figure 4(g)) and cells in the high glucose group (Figure 4(h)), and Nec-1 treatment significantly reversed this phenotype. Therefore, we believe that Nec-1 intervention can reduce proinflammatory cytokine levels in the kidneys of DKD mice.

### 3.5. NAC Treatment Decreased Necroptosis and Inflammation by Inhibiting the RIPK1/RIPK3 Signaling

**Figure 5:**
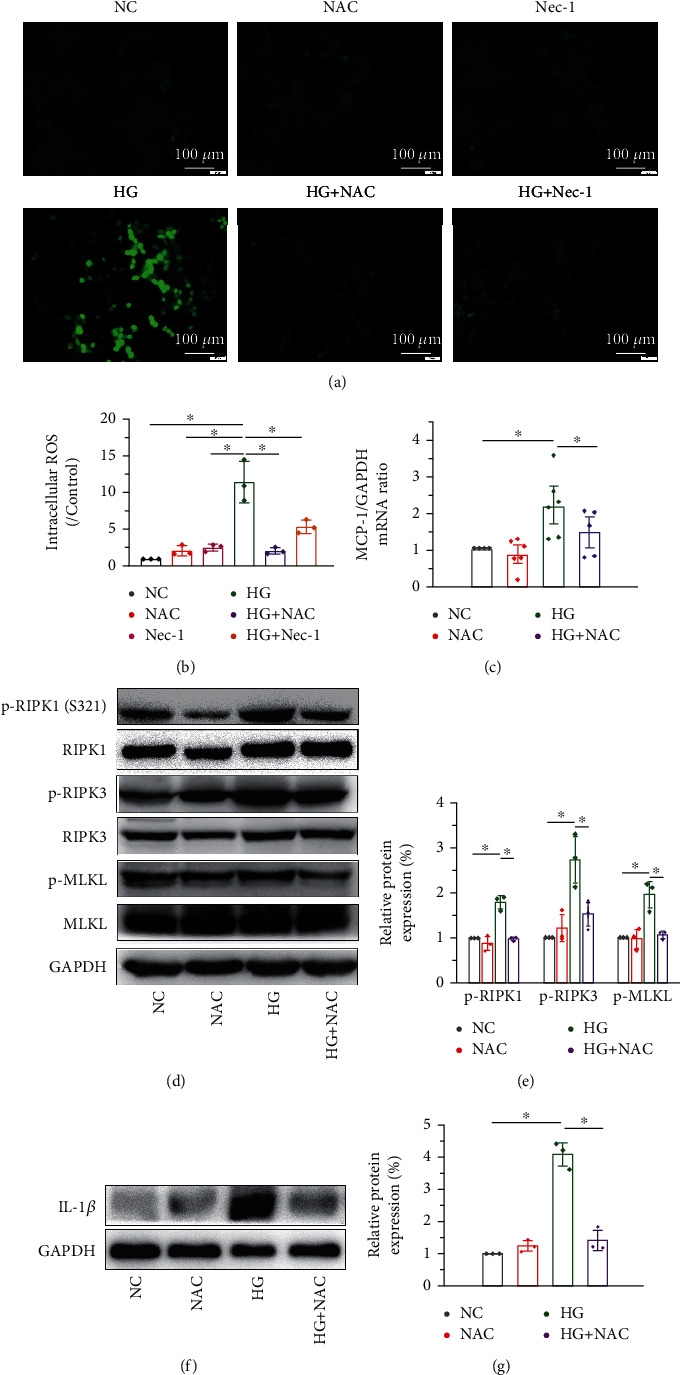
NAC treatment decreases necroptosis and inflammation by inhibiting RIPK1/RIPK3 signaling. (a, b) ROS levels increase significantly upon HG intervention and are effectively downregulated by NAC (2 mM for 48 h) and Nec-1 (50 *μ*M for 48 h). Scale bar: 100 *μ*m. Western blot analysis showing that HG activates the expression levels of the RIPK1 signaling pathway, including p-RIPK1, p-RIPK3, and p-MLKL compared to the control group. The addition of NAC effectively reduces necroptosis of the NRK-52E cells as NAC attenuates the upregulation of p-RIPK1 and p-RIPK3 (d, e). NAC treatment reduces the elevated mRNA level of MCP-1 induced by HG (c) and significantly decreases the level of proinflammatory cytokines (IL-1*β*) in vitro (f, g).

Previous studies have found that the overproduction of ROS plays an important role in programmed cell death, such as apoptosis [37], pyroptosis [38, 39], and necroptosis [40]. Among the various stimuli that trigger necroptosis, ROS are essential factor [41]. However, whether ROS increase kidney necroptosis in DKD remains unclear. Our results supported that HG intervention increased the intracellular ROS levels compared with the control group. Moreover, Nec-1 directly inhibited the elevated ROS, and the effect was equivalent to the treatment with NAC, an inhibitor of ROS (Figures 5(a) and 5(b)). We further explored whether ROS are crucial for HG-induced necroptosis in NRK-52E cells using the antioxidant NAC to inhibit ROS production. Addition of NAC effectively reduced HG-induced necroptosis in NRK-52E cells, as NAC attenuated the upregulation of the RIPK1 signaling pathway, including p-RIPK1, p-RIPK3, and p-MLKL, compared to that in the control group (Figures 5(d) and 5(e)). Meanwhile, NAC treatment significantly lowered the elevated levels of MCP-1 (Figure 5(c)) and significantly downregulated the production of proinflammatory cytokines (IL-1*β*) in vitro (Figures 5(f) and 5(g)). Together, these results highlight the role of ROS in the regulation of HG-induced NRK-52E cell necroptosis via a positive feedback loop involving RIPK1/RIPK3 and confirm that NRK-52E cell necroptosis, at least in part, promotes the generation of ROS.

## 4. Discussion

The mechanisms underlying DKD have not been fully elucidated. At present, studies have found that high glucose [31, 42, 43] and LPS [44] could induce necroptosis and participate in the development of diabetes and related complications. High glucose [32] and AGE [45] levels induce necroptosis in cardiomyocytes, thus contributing to the occurrence and development of diabetic myocardial fibrosis. Hyperglycemia-induced necroptosis of endothelial cells may accelerate the formation of atherosclerotic plaques in diabetes patients [34], and the apoptosis or necroptosis of podocytes [43] and glomerular endothelial cells [42] results in diabetic glomerulopathy and fibrosis. Herein, we found that the expression levels of p-RIPK1/RIPK1, p-RIPK3/RIPK3, and p-MLKL/MLKL in the kidney tissues of DKD mice were significantly increased, suggesting that necroptosis may be involved in the development of DKD. Furthermore, we found that treatment with RIPK1 inhibitor, Nec-1 significantly inhibited the activation of the RIPK1/RIPK3/MLKL signaling pathway, ameliorated renal dysfunction, and reduced kidney inflammation and renal fibrosis. These conclusions indicate the involvement of necroptosis-associated mechanisms in HG-treated kidneys.

Necroptosis is triggered by the ligation of death receptors or initiators, leading to the phosphorylation of RIPK1. This initiates the phosphorylation of a pseudokinase substrate MLKL, which recruits p-RIPK3 to form a necrosome and translocates to the cell membrane, finally leading to necroptosis. Previous studies have revealed important functions and regulatory mechanisms of RIPK1 in inflammation and have indicated that RIPK1 may be a therapeutic target for multiple human diseases [46]. However, our study is the first to show that blocking RIPK1 by Nec-1 ameliorates renal dysfunction and reduces kidney inflammation in DKD. Our findings suggested that Nec-1 significantly reduced necrosis of kidney tissue, decreased the expression of necroptosis markers, and blocked/reduced inflammation. Meanwhile, the activation of RIPK1 and RIPK3 was suggested to play a key role in the process of regulated necrotic cell death [18, 41]. Tumor necrosis factor receptor type 1-related death domain protein (TRADD) acts as an interaction partner of RIPK3 to mediate RIPK1-independent necroptosis [47]. RIPK3-dependent calcium/calmodulin-dependent kinase (CaMKII) activation plays a key role in necroptosis in cardiac ischemia-reperfusion injury models [48]. Thus, the molecular mechanisms underlying necroptosis are complex and diverse. While our research focused on the mechanisms involving RIPK1, further research is required to investigate whether necroptosis induced by high glucose is dependent on the activation of RIPK3 and to validate its role in necroptosis in DKD though knockdown experiments or by the use of specific inhibitors.

Chronic persistent inflammation plays an important role in the progression of DKD. The activation of necroptosis can strongly promote inflammation by regulating the production of inflammatory cytokines and the release of DAMP-related molecular patterns (DAMPs) from damaged cell membranes after lysis [15]. Studies have revealed that RIPK1 was involved in regulating the production and activation of proinflammatory factors such as TNF, IL-6, NLRP3 inflammasome, and NF-*κ*B [49]. RIPK1 plays an important role in the signaling pathways triggered by death receptors through its regulation of caspase-dependent apoptosis and RIPK3/MLKL-mediated necroptosis. It is also involved in promoting cell survival and inflammation by activating the NF-*κ*B pathway [19, 50]. Deubiquitination of RIPK1 promotes the formation of complex IIa with caspase-8 and FADD to induce apoptosis, or complex IIb with RIPK1 and RIPK3 to induce necroptosis [51, 52]. When RIPK1 is modified by ubiquitination, it forms a scaffold for stabilization of IKK*α*/*β*, degradation of I*κ*B*α*, release of NF-*κ*B, and activation of inflammation and prosurvival genes [53]. Given this, we found that the expression of p-I*κ*B*α* and p-IKK*α*/*β*, phosphorylation of S321 RIPK1, and proinflammatory cytokines IL-1*β* and MCP-1 increased. Moreover, inhibition of RIPK1 with Nec-1 effectively lowered the elevated levels in vivo and in vitro. Since the phosphorylation of S321 RIPK1 and IKK*α*/*β* is both mediated by TNF-mediated signaling [54–56], these results suggest that high glucose levels increase the activation of RIPK1-kinase-/NF-*κ*B-dependent inflammation upon stimulation by TNF-*α*. Thus, RIPK1 is the key checkpoint that determines cell survival based on the activation of NF-*κ*B prosurvival signaling or induction of necroptosis.

**Figure 6:**
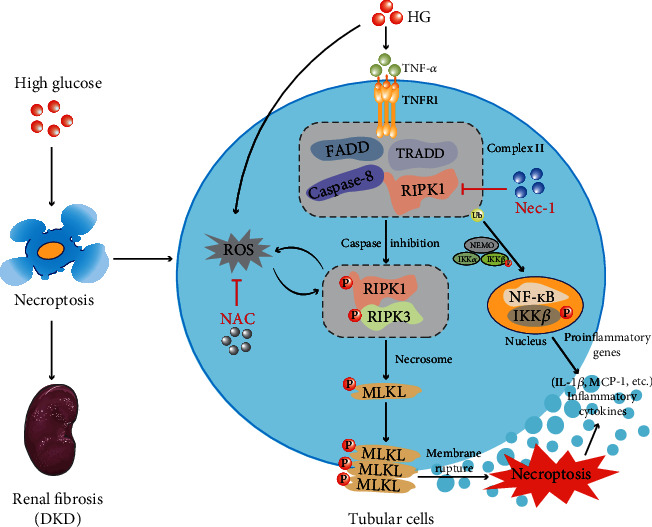
HG intervention drives the progression of DKD by increasing tubular cell necroptosis and inflammasome activation. Stimulated by HG, FADD and TRADD recruit RIPK1 and form complex II when caspase-8 is inhibited. ROS leads to the activation and phosphorylation of RIPK1 to an extent, recruits the RIPK1-RIPK3-MLKL complex, and localizes on the cell membrane via MLKL phosphorylation, ultimately disrupting the plasma membrane and causing cell lysis. When RIPK1 is modified by ubiquitination, it forms a scaffold for the stabilization of IKK*α*/*β*, leading to the release of NF-*κ*B and activation of inflammation and prosurvival genes. Ultimately, occurrence of tubular cell necroptosis, inflammation, renal fibrosis, and renal dysfunction contribute to DKD. HG: high glucose; TNFR1: tumor necrosis factor receptor 1; FADD: Fas-associated protein with death domain; TRADD: TNFR1-associated death domain; NEMO: nuclear factor kappa B essential modulator; IKK*α*/*β*: inhibitor kappa B kinase *α*/*β*; NF-*κ*B: nuclear factor-*κ*B; Ub: ubiquitination; ROS: reactive oxygen species; Nec-1: necrostatin-1; NAC: antioxidant N-acetylcysteine.

ROS are mainly derived from the electron transfer process during mitochondrial oxidative respiration. At the physiological level, ROS are involved in a variety of intracellular signal transduction pathways and play an important role in the regulation of cell proliferation, differentiation, and other physiological processes [57]. Excessive intracellular ROS production leads to an imbalance in oxidative and reductive capacity, and oxidative stress damage occurs in the development of numerous diseases, including cardiovascular system diseases such as coronary heart disease, chronic lung diseases, pulmonary fibrosis [58], neurodegenerative diseases [59], and chronic kidney diseases such as diabetic nephropathy [60]. ROS overproduction leads to oxidative stress injury and cell death in renal tissues, which is one of the key mechanisms underlying the occurrence and development of DKD. In addition, ROS overproduction is involved in mediating programmed necrosis induced by inflammatory factors such as TNF-*α* [61], ischemia-reperfusion injury [48], and respiratory syncytial viral infection [62]. ROS have been considered as a driving force for necroptosis [63], and their major function is the regulation of RIPK1 kinase activity. Three crucial cysteines (cysteines 257, 268, and 586) sense ROS levels and promote RIPK1 autophosphorylation on serine residue 161 (S161) and form a functional necrosome [64]. TNF induced ROS activation in a positive feedback to enhance necrosome formation and induced NRK-52E necroptosis; ROS production decreases upon knockdown of RIPK1 or RIPK3 [65]. However, most of the research on ROS and disease, especially high glucose-stimulated ROS production, did not clearly distinguish the specific type of ROS. Unfortunately, we did not perform a direct analysis to distinguish between various ROS or precisely the specific type of ROS in our study. In our study, we found that HG treatment increased the production of intracellular ROS and the necrosome complex. Furthermore, NAC markedly suppressed necroptosis and proinflammatory cytokine production in NRK-52E, and Nec-1 treatment effectively reduced the elevated ROS levels. Altogether, hyperglycemic necroptosis partly depends on ROS generation, and high glucose can induce necroptosis by promoting ROS overproduction (Figure 6).

## 5. Conclusion

In conclusion, our findings provide the first evidence that Nec-1 inhibits RIPK1/RIPK3/MLKL-dependent signaling by targeting RIPK1 and that NAC partly decreases the activation of necroptosis by preventing the production of ROS, ultimately regulating renal necroptosis, oxidative stress, and inflammation. Consequently, we postulated that the efficacy of Nec-1 or NAC treatment against DKD may be driven by the inhibition of necroptosis signaling. Our data highlights the role of RIPK1 as a key determinant of whether the cell activates the NF-*κ*B prosurvival signaling or undergoes death by necroptosis. Thus, our study underscores RIPK1 as a potential therapeutic target for DKD.

## Data Availability

The data that support the findings of this study are available from the corresponding authors upon reasonable request.
